# Analysis of the role of the Hippo pathway in cancer

**DOI:** 10.1186/s12967-019-1869-4

**Published:** 2019-04-08

**Authors:** Yanyan Han

**Affiliations:** Department of Pathology, Okayama University Graduate School of Medicine, Dentistry, and Pharmaceutical Sciences, 2-5-1, Shikata-cho, Kita-ku, Okayama, 700-8558 Japan

**Keywords:** Hippo pathway, YAP/TAZ, Cancer

## Abstract

Cancer is a serious health issue in the world due to a large body of cancer-related human deaths, and there is no current treatment available to efficiently treat the disease as the tumor is often diagnosed at a serious stage. Moreover, Cancer cells are often resistant to chemotherapy, radiotherapy, and molecular-targeted therapy. Upon further knowledge of mechanisms of tumorigenesis, aggressiveness, metastasis, and resistance to treatments, it is necessary to detect the disease at an earlier stage and for a better response to therapy. The hippo pathway possesses the unique capacity to lead to tumorigenesis. Mutations and altered expression of its core components (MST1/2, LATS1/2, YAP and TAZ) promote the migration, invasion, malignancy of cancer cells. The biological significance and deregulation of it have received a large body of interests in the past few years. Further understanding of hippo pathway will be responsible for cancer treatment. In this review, we try to discover the function of hippo pathway in different diversity of cancers, and discuss how Hippo pathway contributes to other cellular signaling pathways. Also, we try to describe how microRNAs, circRNAs, and ZNFs regulate hippo pathway in the process of cancer. It is necessary to find new therapy strategies for cancer.

## Overview of the Hippo pathway

### Hippo signaling pathway

**Fig. 1:**
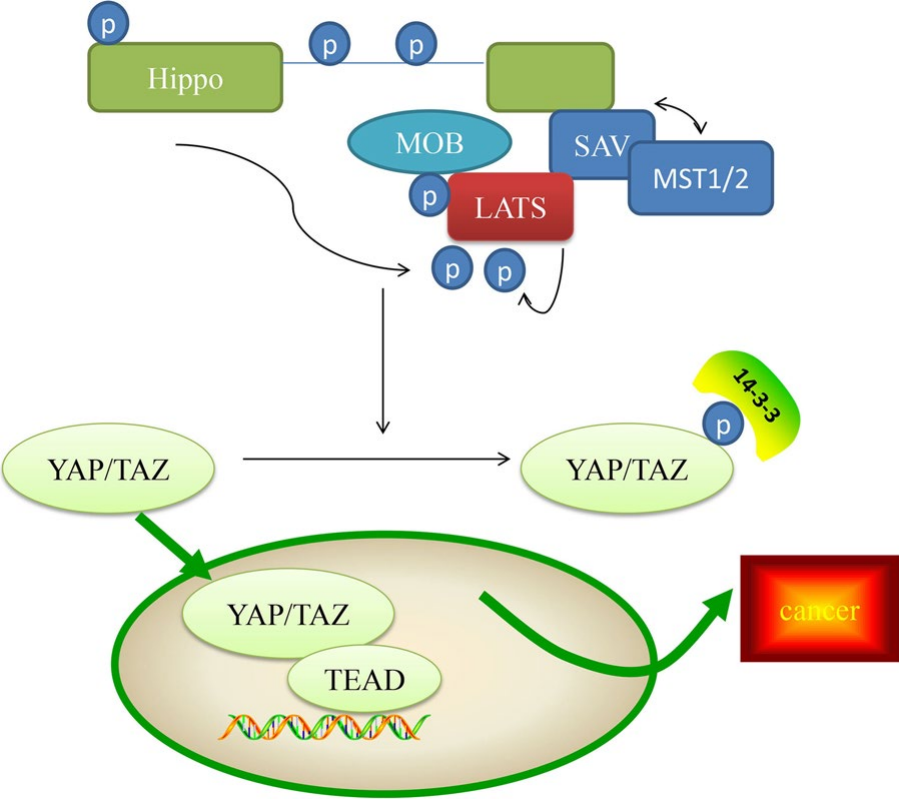
Hippo pathway Phosphorylated MOB recruits LATS (together with SAV and MST1/2). LATS is then phosphorylated by hippo to generate activated LATS, which can then phosphorylate YAP/TAZ and promote their cytoplasmic localization by creating a binding site for 14-3-3 proteins. YAP/TAZ and TEAD, the heterodimer, can enhance cancer

Hippo signaling exerts a critical role in modulating cell proliferation and has been demonstrated to contribute to the progression of various diseases including cancer. The Hippo signaling pathway is primarily composed of mammalian Ste20-like kinases 1/2 (MST1/2) and large tumor suppressor 1/2 (LATS1/2), yes association protein (YAP) and/or its paralog TAZ (which is also known as WW domain containing transcription regulator 1 (WWTR1) [1]. Following the activation of the Hippo pathway, MST1/2 is phosphorylated and activates LATS1/2, which can then phosphorylate YAP/TAZ, resulting in the inhibition of activity of YAP/TAZ. LATS kinases and its mammalian homologs MST1 and MST2 are activated by Hippo (Fig. 1) [1]. There are multiple functions of Hippo kinase activity associated with the activation of Hippo, including the promotion of MOB-Hippo or MOB-LATS binding, and the activation of LATS [2]. MOB proteins have diverse range of roles in the activity of LATS. The ability of MOB proteins to interact with Hippo and LATS kinases can result in the phosphorylation of LATS, and their associations with LATS induce a necessary conformational change [3]. SAV was observed to link Hippo and Warts by acting as a scaffolding protein. In addition, SAV1 can inhibit the recruitment of SLMAP and maintain the activation of MST1/2 [4]. It has previously been reported that MST1/2 kinases can interact with the adaptor protein SAV and with phosphorylated LATS1/2 kinase. The phosphorylation of LATS promotes their cytoplasmic localization in YAP proteins (Ser 127 of human YAP1, Ser 89 of human TAZ) by creating a binding site on 14-3-3 proteins [2]. In addition, previous studies have demonstrated that the expression of YAP/TAZ is unusually excessive in tumors, enhances the occurrence of tumors and is thought to be a cancer gene for a large number of solid cancers [5, 6].

### YAP proteins

**Fig. 2:**
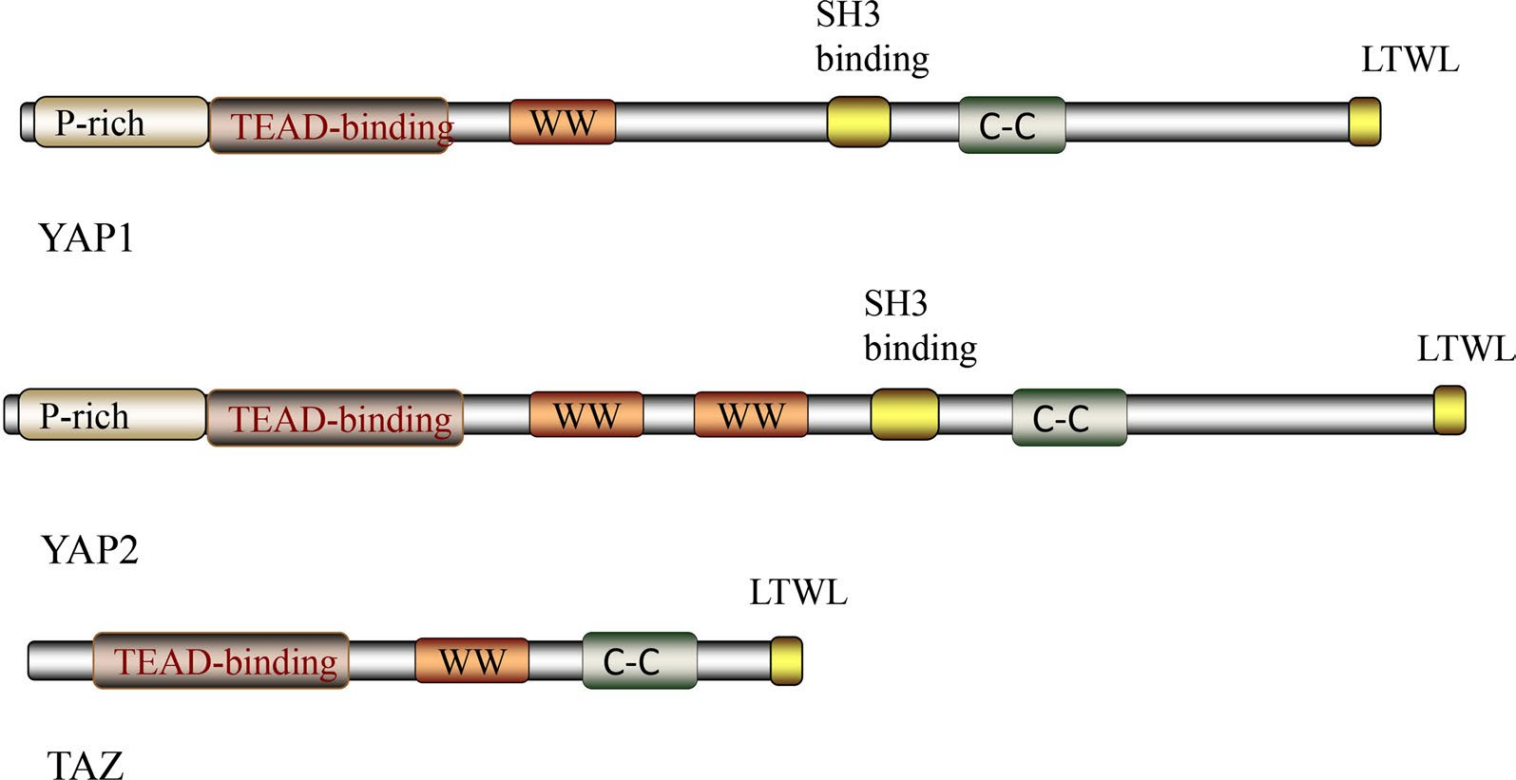
YAP protein and TAZ protein YAP protein is involved in YAP1 and YAP2 proteins. YAP1, it is involved in one WW domain, whereas, YAP2 composes of two WW domains. TAZ isoform that is commonly studied possesses one WW domain. In contrast with YAP, TAZ lacks of p-rich and SH3-binding domains

Hippo signaling can influence cellular phenotypes by inhibiting the transcriptional co-activator protein Yki, or its mammalian homologs YAP and TAZ [7]. Several DNA-binding partners have been identified, which are essential for the activation of the YAP protein, as YAP lacks a DNA-binding domain. Of these partners, the primary ones are TEAD proteins, including TEAD 1–4 of mammals, and Scalloped (Sd) of flies [8]. The WW domain has two highly conserved tryptophans, and has been hypothesized to modulate protein–protein interactions, and the other WW domain is conserved in YAP across species. It has been reported that YAP1contains only one WW domain, whereas, YAP2 consists of two WW domains [1] (Fig. 2). A previous study performed a binding assay and observed that the WW domains can associate with PPXY motifs of WBP-1 and WBP-2 [9]. Of note, YAP was identified to be a transcription co-activator containing a C-terminal transcription activation domain [10]. The four amino acids of the C-terminal of YAP interact with PDZ domains in proteins, and may be important in regulating the subcellular localization of YAP [11]. A recent study demonstrated that the YAP-TEAD heterodimer could interact with other transcription factors involved in Taiman, GAGA, AP-1, and β-catenin, and contribute to the regulation of downstream genes [12].

The phosphorylation of YAP on Ser127 binds with the 14-3-3 protein and inhibits its transcriptional activity via cytoplasmic sequestration. A phosphatase, such as protein phosphatase-1, can dephosphorylate YAP Ser127 and enhance the nuclear accumulation and transcription activity of YAP [13]. The phosphorylation of on YAP Ser381, can affect its stability and induce subsequent phosphorylation by casein kinase 1, and activate a phosphodegron degradation motif [14].

Growth promoting genes such as Myc, CycE and E2F1, which are cell cycle regulators, and Diap1 and BIRC3, which are inhibitors of apoptosis, contribute to the activation of YAP proteins, which is associated with an increase in tissue growth [15]. The ligands of the Wnt, Notch, EGFR, TGF-β, and JAK-STAT signaling pathway have also been identified as targets of YAP proteins, which can regulate tissue growth [16]. In addition, a group of upstream components of the Hippo pathway, including Merlin, Ex, Kibra, Angiomotin-like 2 (AMOTL2), and LATS kinases have been reported to serve an essential role in negatively coordinating YAP proteins [1].

### TAZ protein

The structure of TAZ is also composed of two tryptophan residues [17]. The commonly observed human TAZ isoform possesses one WW domain (Fig. 2). TAZ also shares a C-terminal PDZ-binding motif, which mediates interactions with the 80–90 amino acid protein-interaction domains. PDZ domains [18] have been identified in several proteins, many of which are associated with trans-membrane or cytoskeleton. Additionally, a domain in the N-terminal region of TAZ mediates its binding to TEAD transcription factors. Although much of the TEAD-binding domain is conserved in TAZ, recent molecular modeling studies have confirmed differences in the combination of TAZ and YAP and TEAD transcription factors, due to TAZ lacking the PxxΦP motif [19]. The TEAD-binding region in TAZ is in very close interaction with that required for 14-3-3 binding, which fosters cytoplasmic sequestration and is one of the major mechanisms through which the Hippo pathway controls TAZ/YAP localization and activity.

Although TAZ and YAP shared several identical features, differences are evident. For example, the extreme N-terminus of YAP contains a proline-rich region; however, this has not been identified in TAZ. It has been reported that the proline-rich area interacts with the heterogeneous nuclear ribonuclear protein U (hnRNPU), which serves a role in mRNA processing [20]. YAP also possesses a SH3-binding motif (amino acids PVKQPPPLAP), which is lacking in TAZ. This region mediates interactions between the SH3 domains of several proteins, including the YES and SRC kinases, as well as the adaptor proteins NCK and CRK [21]. Despite the aforementioned examples, differences in the features of TAZ and YAP have not been the subject of much investigation, and require further study.

## Signals of regulation of Hippo signaling

**Fig. 3:**
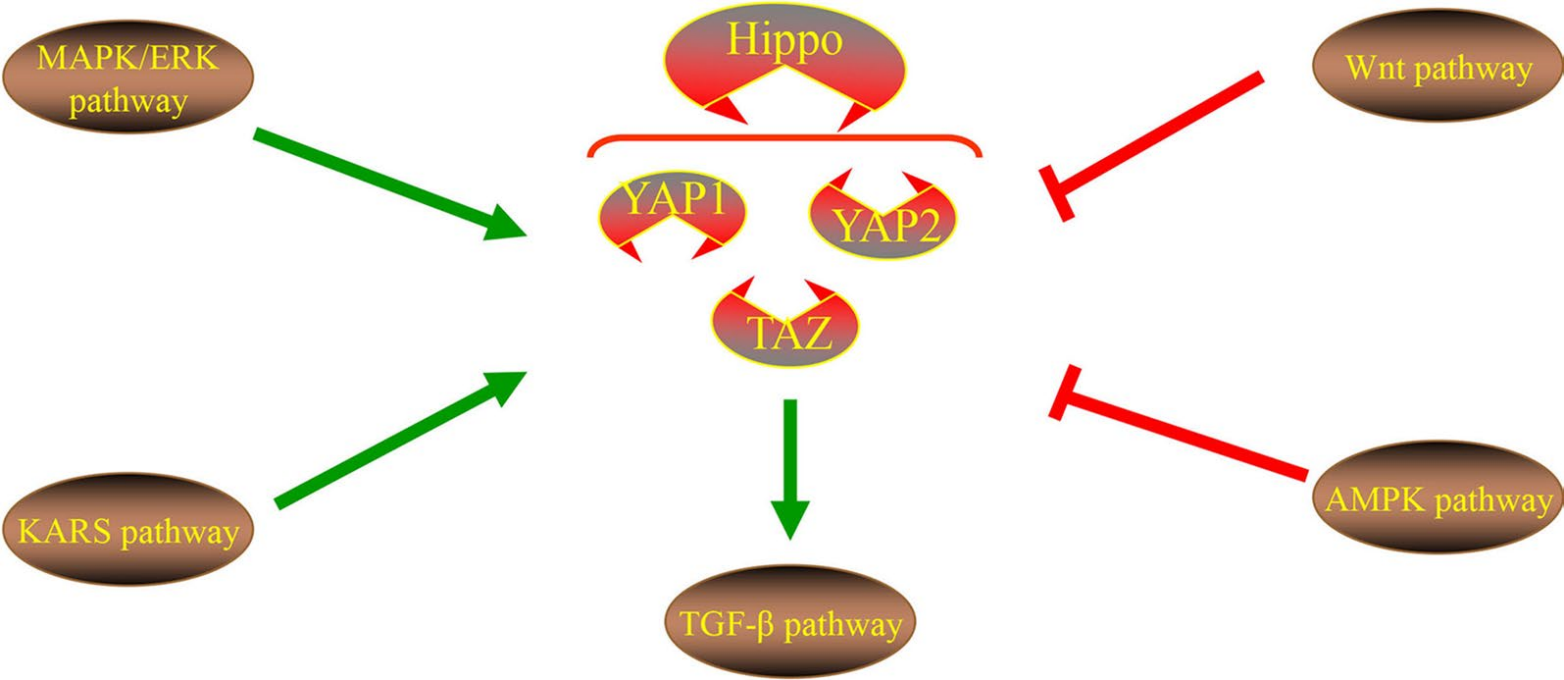
Different pathways regulation of hippo pathway. Wnt pathway and AMPK pathway inhibit YAP protein. TAZ protein promotes TGF-β pathway. KRAS and MAPK/ERK promotes YAP protein

Hippo signaling can be modulated by various signals in cancer cells (Fig. 3), which exert a critical role in tumorigenesis. Therefore, it is imperative to observe this signaling network in order to improve the presently available understanding.

### Wnt pathway

Overexpression of the Wnt pathway possesses frequent APC or β-catenin mutations, which is a key feature in human cancer. In terms of scaffolding proteins such as APC, Axin and DVL of canonical Wnt signaling can act as key intracellular regulators of β-catenin dynamics [22]. An abundance of studies have concentrated on the role of YAP in Wnt suppression by DVL, through which YAP cannot tightly associate with Hippo and Wnt [23]. In addition, the scaffolding protein of DVL drives the nuclear export of the phosphorylation YAP. The DVL contains conserved nuclear export signals (NES, M/LxxLxL) located next to the DEP domain, with NES being responsible for the cytoplasmic translocation of YAP. Importantly, the cytoplasmic translocation of YAP is induced by the close contact inhibition of E-cahderin, α-catenin or AMPK activation, which requires DVL. This indicates that the Wnt scaffold protein DVL exerts a key shuttle effect on the Hippo pathway. In summary, the function of the Wnt pathway is opposite to that of the YAP protein, which may provide the basis for an important therapeutic strategy.

### AMPK pathway

AMPK can act as a key cellular energy sensor to directly phosphorylate YAP at multiple sites, and as a primary cellular metabolism regulator to impair its activity. The hyper-expression of AMPK suppresses cell proliferation, and YAP and TAZ, both of which can result in the inhibition of AMPK-mediated growth [24]. In addition, AMPK may also indirectly phosphorylate AMOTL1, which can promote the activity of LATS as well inhibiting the activity of YAP and inducing YAP phosphorylation, further strengthening the AMPK-mediated inhibitory regulation on YAP [25]. Additionally, LKB1 has been demonstrated to inhibit Yki activity in an AMPK-dependent manner in a Drosophila study, although other mechanisms were also observed in human cells [26]. Notably, cellular energy stress can inhibit YAP through AMPK-dependent and -independent mechanisms. In AMPK knockout cells, LATS kinase can be activated to cope with energy stress, suggesting that cell energy stress can also activate the hippo pathway through mechanisms unrelated to AMPK. Glucose starvation activating molecular basis of LATS is an interesting hypothesis that requires further investigation [27]. Based on these perspectives, the association between AMPK and YAP require further invesitgation.

### TGF-β pathways

The TGF-β signaling pathway participates in various cellular functions including cell proliferation, apoptosis, differentiation and remodeling of the extracellular matrix [28]. Previous studies have detailed the relationship between TAZ/YAP and TGF-β through analyzing the levels of genes associated with intracellular signaling and transcriptional regulation [29]. TAZ binds to heteromeric Smad2/3-Smad4 complexes and is necessary for TGF-β response elements, resulting in maintaining the nuclear accumulation of the Smad2/3-Smad4 complex and TGF-β mediated stem cell self-renewal [30]. The cytoplasmic retention of phosphorylated TAZ can prevent Smad2/Smad4 complexes from accumulating in the nucleus, while TAZ knockout can lead to TGF signal inhibition and neural epithelial differentiation [31]. Smad2/3, TEAD4, and TAZ/YAP form a complex with OCT4, which promotes the transcription of pluripotency genes while suppressing mesendodermal differentiation. Through the co-regulation of the Smad2/3-Smad4 complex and FOXH1, the disruption of this complex was observed to promote the induction of mesendodermal genes. Therefore Smad2/3-mediated transcription seems to be a positive and negative regulator of interactions with TAZ/YAP in a context-dependent manner [29].

### KRAS signaling

Recent observations have indicated that the overexpression of YAP1 dissociates K-Ras4B inhibition. Therefore, the transcriptional activation of YAP1 and β-catenin as a survival rescue strategy of K-Ras4B-inhibited cells [32, 33] has further become a key concern, as it can negate the effects of K-Ras4B therapeutics. YAP1 and β-catenin transcriptional regulators serve a role in the targeting of K-Ras4B and can induce resistance by controlling the progression of cells from the G1 phase to S phase in the cell cycle. The role of ERK corresponds to that of YAP1 and PI3Kα. ERK and YAP can produce consequences similar to those of PI3K and β-catenin, which is why K-Ras4B drug resistance is caused by the expropriation of YAP and β-catenin [32–34]. The emerging picture from relevant experimental and clinical data suggests that oncogenic KRAS, YAP1 and β -catenin serve similar roles in cell cycle control in tumor initiation [34].

### MAPK/ERK signaling

Recent studies have reported that mutations in the Hippo signaling components SAV1, MST1/2 and Lats1/2 are not evident in human cancer. Even if rendering this signaling, it can unlikely explain the vigorous activation of the YAP in the majority of; however, not all human tumors. An associated study has also demonstrated that an activated YAP promotes the resistance to MAPK/ERK Kinase (MEK)-targeted inhibitor therapy [35]. The sensitivity of YAP elevated cancer cells to MEK inhibitors and the knockdown of this effect has been demonstrated in numerous types of cancer cells. Therefore, YAP activity predicts the therapeutic effect of MEK inhibitors in patients with cancer with activated MAPK signals. Additionally, previous studies have demonstrated in comparable MAPK vs. YAP1 cell cycle actions, that the nucleotide sequences of the DNA response elements of the downstream transcription factor complexes modulated by these two cellular pathways are similar [36, 37].

## Role of microRNAs on Hippo pathway in cancer

### microRNAs

**Fig. 4:**
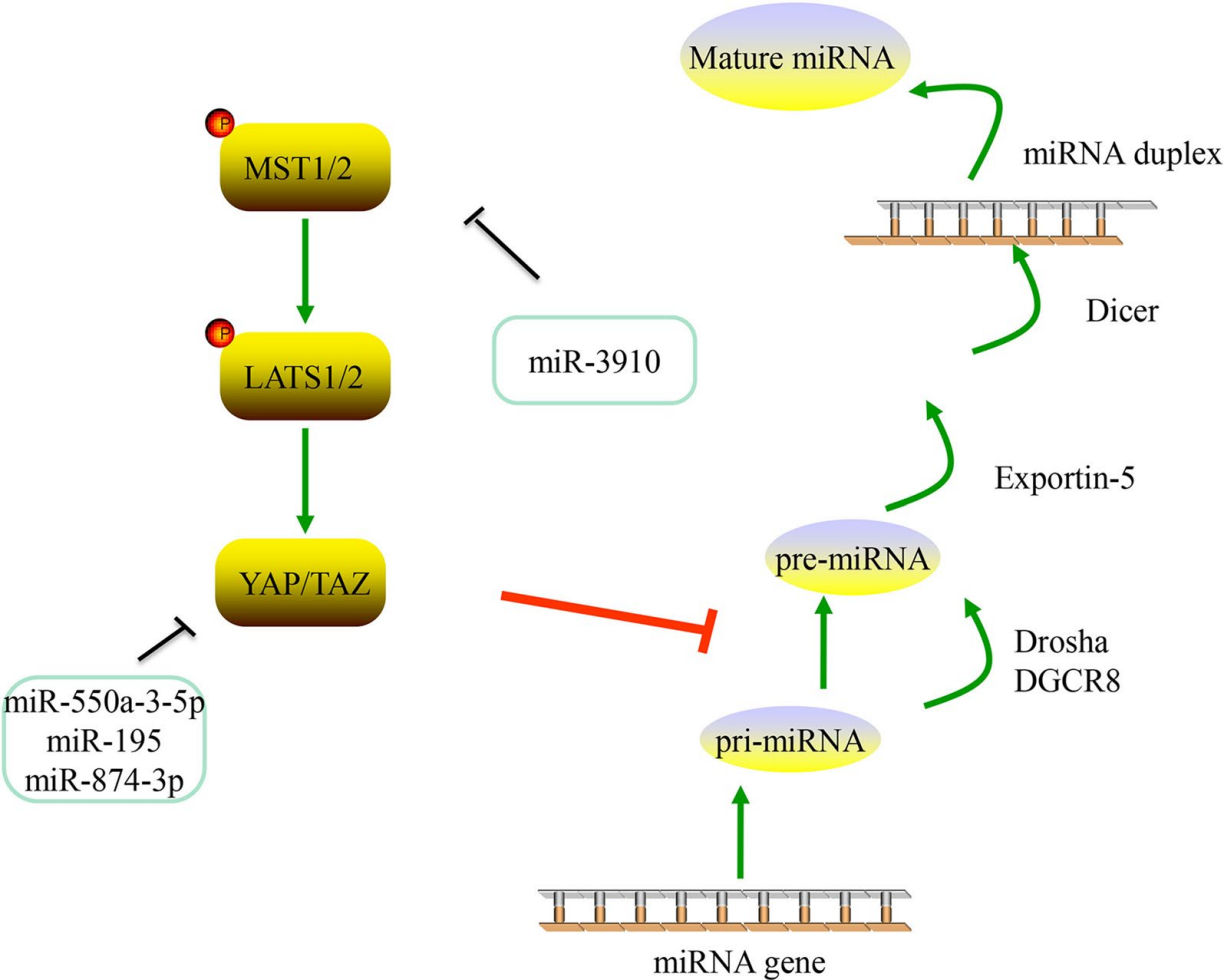
miRNAs and Hippo pathway. YAP/TAZ could inhibit the processing of pri-miRNA to pre-miRNA. Several miRNAs can make an effect on Hippo pathway

microRNAs (miRNAs) are a group of small regulatory RNAs that function in the 3′untranslated region (UTR) of a messenger RNA and guide post-transcription inhibition by binding in a sequence-specific manner [38]. The biogenesis of miRNAs begins with the transcription of RNA polymerase II from the miRNA gene to the primary miRNA (pri-miRNA), which has been demonstrated to form a hairpin loop [39]. The end of the hairpin is then recognized and processed into a 60-nt stem-loop structure called a pre-miRNA by the endonuclease Drosha and DGCR8. The pre-miRNA is then exported out of the nucleus via Exportin 5 and RAN-GTP, where it is further processed into the mature miRNA [40]. The mature miRNA is located to an Argonaute protein with assistance from additional chaperone proteins in a complex known as the RNA-induced silencing complex (RISC) [41]. The RISC-associated miRNA is then guided by the “seed region” (nucleotides 2 to 8) to the complementary seed match” site of a target mRNA, resulting in the degradation of mRNA [42]. Importantly, the seed area of any particular miRNA is expected to target thousands of mRNA, and each mRNA can have hundreds of miRNA seed matching sites in their 3′UTR [42]. Due to this broad regulatory potential, miRNAs are considered to be the largest type of gene regulator capable of “fine-tuning” gene expression, and influencing the pathogenesis/progression of a variety of diseases [43]. microRNAs can affect cancer through the hippo pathway, which was described below and summarized in Fig. 4 and Table 1.

**Table 1:** microRNAs in cancers

microRNAs	Function	Cancer	Mechanism	References
miR-550a-3-5p	Suppressed cell proliferation, metastasis, and tumor sphere formation	Colon, esophageal,melanoma	Inhibiting YAP	[45]
miR-665	Promoted cell proliferation, migration, invasion, and the epithelial–mesenchymal transition	Hepotacellular	Inhibiting hippo pathway	[47]
miR-195	Inhibited the migration, invasion and epithelial–mesenchymal transition	Gastric, breast, thyroid and hepotacellular	Suppressing YAP	[50]
miR-3910	Inhibited apoptosis induced the tumor formation	Hepotacellular	Suppressing MST1	[53, 54]
miR-874-3p	Increased the apoptosis ratio and decreased the mitochondrial potential	Colorectal	Inhibited YAP and TAZ	[55]

### microRNAs and Hippo pathway

#### miR-550a-3-5p

Recent data has demonstrated that miR-550a-3-5p suppresses cell proliferation, metastasis, and tumor sphere formation through directly inhibiting oncogenic YAP in various types of cancer cells [44]. Additional studies have also demonstrated that miR-50a-3-5p-mediated YAP regulation has clinical correlations in a variety of cancers, including the inverse relationship between the signature of miR-50a-3-5p and YAP in colon cancer, and its prognostic value in esophageal cancer. Importantly, Choe et al. reported that treatment with miR-550a-3-5p improved the sensitivity of vemurafenib by inhibiting YAP, and reducing the activity of AKT in vemurafenib-resistant colon cancer and melanoma cells [45].

#### miR-665

Previous studies have demonstrated that the expression of mir-665 in hepotacelluar carcinoma (HCC) and tissues is elevated [46]. The patients with HCC with a high expression of mir-665 exhibited more severe tumor size, vascular infiltration and Edmondson grading. Functional loss or gain tests have demonstrated that mir-665 promotes the proliferation, migration, invasion and epithelial-mesenchymal transition (EMT) of HCC cells in vitro and in vivo. Tyrosine phosphatase receptor type B (PTPRB) was observed to be downregulated in HCC tissues, and was negatively correlated with miR-665 expression. The restoration of PTPRB reverses the effects of miR-665 on HCC migration and invasion. In addition, a mechanistic study clearly demonstrated that PTPTRB mediated the functional role of miR-665 through regulation of the Hippo signaling pathway. In summary, modulating PTPRB expression reversed the effects of miR-665 on the Hippo signaling pathway [47].

#### miR-195

miR-195 belongs to the miR-15 family. An increasing amount of research has focused on the functions of miR-195 in tumorigenesis over the past few years. miR-195 is considered to function as a tumor inhibitor in certain types of cancer [48, 49]. The study of Yu et al. demonstrated that the expression of miR-195 was markedly decreased in HCC, and its low expression level was closely associated with the poor clinical characteristics in patients with HCC. Conversely, the level of YAP in HCC tissues is extremely high, and the level of YAP in metastatic tissue is equally high. In addition, a strong negative correlation was identified in HCC tissues with low levels of miR-195 expression and high levels of YAP. Notably, this study confirmed that significant predictors of prognosis can be used in patients with HCC, including miR-195, YAP and their combinations, and mechanistically confirmed that miR-195 inhibits the migration, invasion and EMT of HCC cells, which is related associated with the repression of YAP [50].

#### miR-3910

miR-3910 has been observed to promote the growth of HCC cells on liquid culture and soft agar. In addition, miR-3910 is a positive regulator of cellular dynamics, as evidenced by a Boyden chamber assay and an intrahepatic metastasis assay. These observations clearly demonstrated the carcinogenic effects of miR-3910 in HCC tumors. The application of YAP signal activation in the occurrence of HCC has been widely identified [51, 52]. The knockdown of MST1, MST2, and SAV has been demonstrated to induce tumor formation in the liver of mice [53], emphasizing the pivotal function of YAP signaling in the initiation of HCC. The study of Cheng et al. identified that miR-3910 regulated the expression of MST1, saved the apoptosis induced by MST1, and confirmed that miR-3910 exerted a carcinogenic function with MST1 as the target [54].

#### miR-874-3p

The expression of miR-874-3p was significantly decreased in colorectal carcinoma (CRC) tissues compared with adjacent normal tissues. It has also been observed that the upregulation of miR-874-3p increased the apoptosis ratio and decreased the mitochondrial potential of CRC cells with 5-FU treatment in vitro, and reduced the chemoresistance of CRC cells to 5-FU in vivo. By contrast, the knockdown of miR-874-3p decreased the apoptosis ratio and increased the mitochondrial potential of CRC cells following treatment with 5-FU treatment in vitro and enhanced the chemo-resistance of CRC cells to 5-FU treatment in vivo. Que et al. demonstrated that miR-874-3p directly inhibited YAP and TAZ, leading to the inactivation of TEAD transcription and the downregulation of Hippo downstream target genes, such as CTGF, BCL2L1 and cyclin A [55].

## Role of circular RNAs on Hippo pathway in cancer

### circRNAs

circRNAs have originally been considered as non-functional accidental by-products of aberrant splicing [56], and to a certain extent, have been the focus of numerous studies. Due to the emergence of next-generation sequencing techniques, a large number of circRNAs have been identified to be widely expressed in eukaryotic cells. circRNA is a single-chain transcription obtained from exons, introns, or intergenic regions, which have covalently closed continuous loops, display cell or tissue specific expression, and remains constant between species due to resistance to RNase R [57]. Due to the closed structure, circRNAs have been observed to be highly stable. Numerous circRNAs exhibit evolutionary conservation, and the expression spectrum is unique to the cell type or stage of development [58]. The function of circRNAs on the Hippo pathway iss summarized in Table 2.

**Table 2:** Circular RNAs in cancers

Circular RNAs	Function	Cancer	Mechanism	References
circ_104075	Promoted cancer growth	Hepatocellular	Up-regulate YAP expression	[59]
hsa_circ_0023404	Promoted the proliferation and promoted the cell-cycle progression suppressed cell migration and invasion	Colorectal	Activated YAP pathway in CC via promoting TFCP2 expression by sponging miR-136	[60]
circPVT1	An increase of the malignant phenotype	Head and neck squamous cell carcinoma	Binding to YAP	[64, 65]
circLARP4	Inhibits cancer cell migration and invasion	Gastric	Regulation of miR-424/LATS1/YAP signaling pathway	[66, 67]

### circular RNAs and Hippo pathway

#### circ_104075

The expression of circ_104075 was observed to be markedly elevated in HCC tissues, cell lines and serum. circRNA can act as a competing endogenous RNA (ceRNA) to absorb microRNA, and indirectly stimulate protein expression. circ_104075 can also upregulate the expression of YAP by absorbing miR-582-3p by acting as a ceRNA. Of note, an N^6^-methyladenosine (m^6^A) motif was identified in the 353-357 region of YAP 3′UTR, which was revealed to be essential for interactions between miR-582-3p and YAP 3′UTR. In addition, recent study has demonstrated that circ_104075 can be used for diagnosis [59]. In summary, circ_104075 may serve as a potential target for the diagnosis and treatment of HCC.

#### hsa_circ_0023404

circRNA hsa_circ_0023404 was significantly upregulated in CC tissues when compared with adjacent normal tissues, which was indicative of a poor prognosis in patients with CC. Functionally, the knockdown hsa_circ_0023404 significantly suppressed the proliferation, arrested cell-cycle progression and inhibited cell migration and invasion in CC. Mechanistically, hsa_circ_0023404 acted as a sponge of miR-136, which is an activator of the YAP signaling pathway. Additionally, hsa_circ_0023404 activated the YAP pathway in CC via promoting the expression of TFCP2 by sponging miR-136, leading to the development and progression of CC [60].

#### circPVT1

Memczak et al. first identified CircPVT1 as circ6 [61] and then named circPVT1 after its host gene, PVT1, which was analyzed in subsequent studies [62, 63]. The PVT1 gene is highly expressed in numerous types of cancers, such as head and neck squamous cell carcinoma (HNSCC) [64]. The circPVT1 locus is in the long non-coding RNA PVT1, and it originates from exon 2 of the PVT1 gene (human genome GRch38/hg38). circPVT1 is highly expressed in tumors compared with non-tumor tissues, especially in patients with TP53 mutations. YAP can regulate circPVT1 at the post-transcriptional level, and following YAP knockdown, the binding to circPVT1 was lost. In addition, YAP did not bind circPVT1 as a results of the downregulation of of mut-p53 [65].

#### circLARP4

circLARP4 exhibits the potential to sponge miR-424, and recent studies have demonstrated that LARP4 inhibits cancer cell migration and invasion via a La-related RNA-binding protein [66]. The study of Zhang et al. reported that circLARP4 was differentially-expressed between GC and adjacent normal tissues, and was derived from Exon 9, 10 of the LARP4 gene and intermediate long intron. Further functional experiments have demonstrated that the excessive expression of peripheral LARP4 inhibits the synthesis of DNA, cell proliferation and invasion by sponging miR-424 and regulating of the expression of LATS1 and YAP genes. circLARP4 revived LATS1 and p-YAP expression, decreased the expression of YAP, restored the expression of LAST1 and p-YAP, and reduced YAP expression, as well as offset the impact of miR-424., suggesting that circLARP4 may function as a tumor suppressive factor in GC via regulation of the miR-424/LATS1/YAP signaling pathway [67].

## Role of ZNFs on Hippo pathway in cancer

### ZNFs

The first zinc finger (ZNF) was transcriptional factor (TFIIIa) of *Xenopus laevis* [68]. ZNFs are classified based on the zinc-finger domain structure. Relevant research has revealed that there are eight different types of zinc finger motifs including Cys_2_His_2_ (C_2_H_2_) like, Gag knuckle, Treble clef, zinc ribbon, Zn_2_/Cys_6_, TAZ2 domain like, zinc binding loops as well as Metallothionein [69].

The motifs of amino acid residues form mononuclear Zn-binding sites such as ZnHis_2_Cys_2_, ZnHisCys_3_, and ZnCys_4_. The most commonly identified motifs include C_2_H_2_-type, treble clef, and zinc ribbon. RING finger proteins, contain Zn_3_His and ZnCys_4_ sites and serve a pivotal function in the process of ubiquitination, and are unique [70, 71]. The monograph describes in detail the zinc finger (Zf) pattern that binds to DNA, RNA, proteins, and small molecules and considered to be important Zf domains. A shift of the central zinc ion combined with the coordination of mutations in amino acids can lead to the loss of ZFS biological function, a process that can be used to inhibit the activity of ZFS in processes that are dangerous to the human body, especially the occurrence of tumors [72]. Therefore, Zf ZF can be used as a bio-target, which is affected by the purpose of destroying conformation control of the role of zinc. This causes it to be released and triggers a functional interruption in processes like transcription.

Recent findings have highlighted the importance of ZNFs in the onset and progression of cancer through regulating the Hippo pathway. ZNFs are involved in all the principal pathways in which cancer is formed ranging from carcinogenesis to metastasis. Additionally, ZNFs are involved in cancer via their transcription factor function. In addition, emerging evidence suggested the importance of the ZNF protein as a chromatin modifier or as a structural protein that regulates the migration and invasion of cancer cells.

**Fig. 5:**
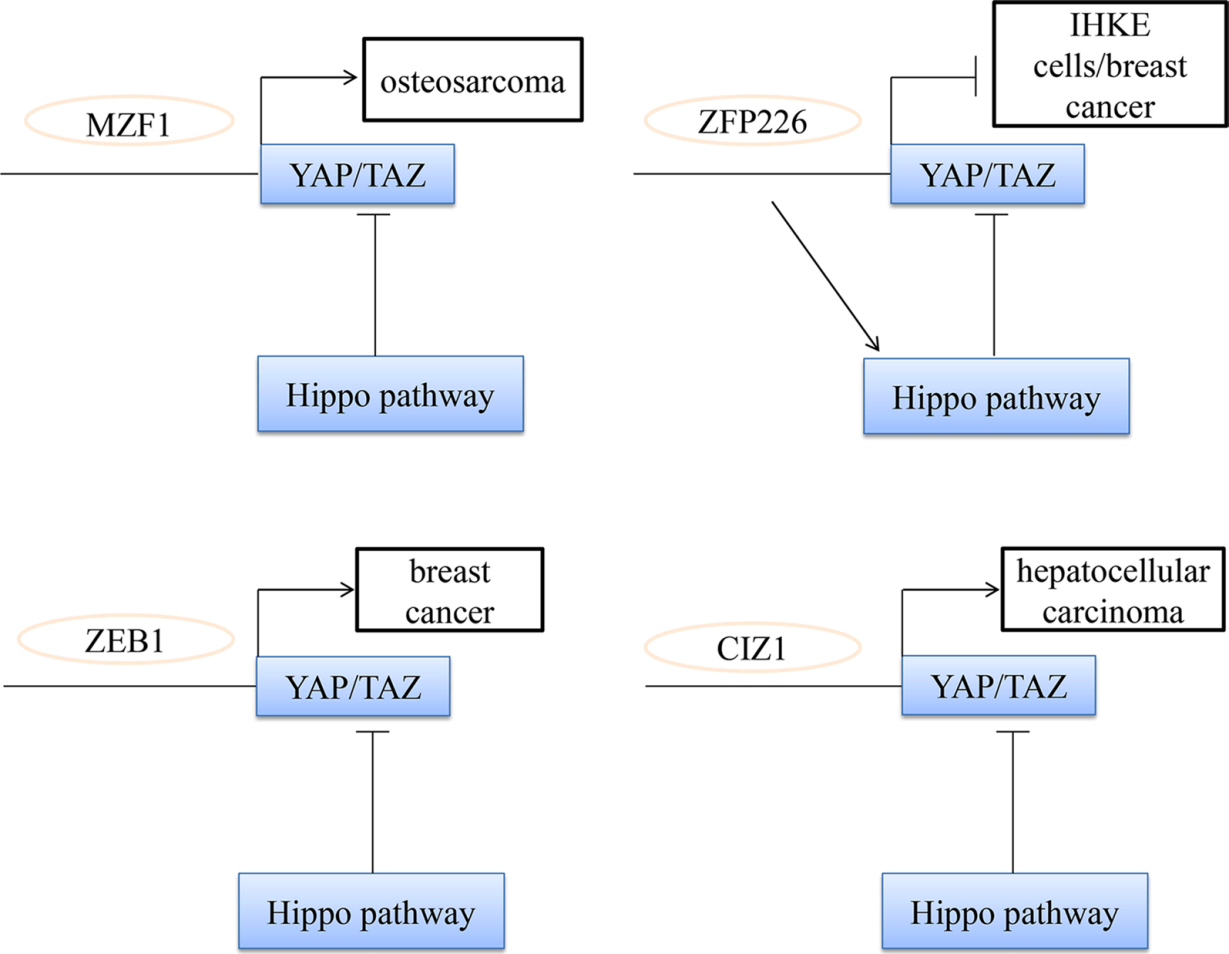
The relationship between zinc finger proteins and hippo pathway in cancers. MZF1 promote osteosarcomas through stimulation of YAP1 expression. ZFP226 was able to induce apoptosis by activating hippo signaling in human breast adenocarcinoma. Cooperation of ZEB1 and YAP stimulates and increases risk in breast cancer. CIZ1 interacted with the transcriptional factor YAP in hepatocellular carcinoma and enhanced cancer growth

Transcription factors serve a central role in regulating gene expression and mediating a set of biological processes, including differentiation, development, metabolism, apoptosis, autologous phagocytosis and the maintenance of stem cells [73]. According to different DNA binding patterns, transcription factors are mainly divided into classical zinc fingers, homeodomains and a basic helix-loop-helix. Among these, classical zinc finger containing proteins (ZNFs) are composed of the largest sequence of specific DNA binding protein families, encoded by 2% of the human gene [74]. Previous studies exhibit different regulatory mechanisms on a variety of downstream genes by recruiting different chromatin modifiers. Specific ZNF proteins are used as transcription inhibitors by recruiting co-pressure proteins [75]. We subsequently described several ZNF proteins in order to evaluate whether they can regulate the hippo pathway and effect the progression of tumors (Fig. 5).

### ZNFs and Hippo pathway

#### MZF1

myeloid zinc finger 1 (MZF1) has been observed to be elevated in several cancers, and its excessive expression inhibits apoptosis and promotes the occurrence of tumors [76], while the silencing of MZF1 leads to reduced tumor growth. Recent data has indicated that MZF1 is essential for the transcription of basal YAP1, and promotes tumor formation through stimulating the expression of YAP1. The factor is highly expressed in osteosarcomas (Oss) and binds to distinct sites in the YAP1 enhancer. Depletion of this factor leads to a drastically reduced YAP1 expression and therefore reverses stem cell properties, as well as producing an opposing effect via downregulating the expression of YAP1, which decreases the stem cell fraction and restores osteogenic differentiation [77].

#### ZFP226

ZFP226 belongs to the Cys_2_–His_2_ zinc finger type and recognizes a nine base pair DNA sequence 5′-GGC-GGC-GGC-3′ in the well-characterized KIBRA core promoter P1a [78]. This DNA target sequence was selected due to its position in a highly sensitive transcription active region, which is highly sensitive to promoter methylation [79]. Previous studies have observed a significant increase in the relative levels of plats 1, and importantly in p-YAP in IHKE cells at 48 h following ZFP226 transfection [78]. Of Note, YAP expression was unaffected by pZFP226 transfection [80]. Most importantly, the study of Schelleckes et al. reported that ZFP226 was able to induce apoptosis by activating hippo signaling in human breast adenocarcinoma cells following transfection with ZPF226.

#### ZEB1

The zinc finger E-box-binding homebox (ZEB) family includes ZEB1 and ZEB2, which are important nuclear transcription factors and have been reported to be key factors in EMT [81]. Mechanistically, ZEB1 can suppress epithelial genes that can stimulate an undifferentiated and highly motile phenotype as a transcriptional repressor [82]. This property of ZEB1 is considered to exert an important role in metastasis, and this has been demonstrated in numerous types of model systems. Lehmann et al. reported that ZEB1 can directly interact with YAP, which can render ZEB1 from a repressor to a transcriptional activator, and thereby act as a similar cancer-promoting effect. In addition, functional cooperation between ZEB1 and YAP is a useful predictor of poor survival, and significantly increases the metastatic risk in hormone receptor-negative breast cancer [83].

#### CIZ1

Previous studies have demonstrated that Cip1 activated YAP signaling in HCC cells through interactions with zinc finger protein 1 (CIZ1), and also induced the growth and migration of cancer cells. The study Liu et al. demonstrated that CIZ1 interacted with the transcriptional factor YAP in HCC cells. CIZ1 can interact with YAP due to its nuclear matrix anchor domain, and CIZ1 also enhanced the YAP to interact with TEAD. Additionally, knocking down CIZ1 decreased the transcriptional activity as well as the biological functions of YAP. Based on these discoveries, CIZ1 is a positive regulator of YAP signaling, and may serve as a therapeutic strategy for HCC [84].

## Function of Hippo pathway in cancers

### Hippo pathway in carcinoma

#### NSCLC

In non-small cell lung cancer (NSCLC), the overexpression of YAP is associated with the development, progression and poor prognosis of diseases, and the TAZ exerts a similar function. An epidemiological study demonstrated that a mutation of YAP, which causes carcinogenicity hyperactivity, is associated with the occurrence of lung cancer [85]. In vivo studies in lung adenocarcinoma mouse models have revealed that the genetic loss of YAP reduces the number of experimentally induced tumor masses in mice [86, 87].

The increase in the nuclear activity of YAP and TAZ may be caused by an increase in signals, receptors or sensors that actively regulate YAP/TAZ activity, or a decrease in signals or molecules that negatively regulate the carcinogenic function of YAP/TAZ [88]. For example, oncogenic ABL1 and 2 kinases have been observed to be overexpressed or hyperactivated in NSCLC [89, 90] and are also involved in the tumor growth and metastasis of lung cancer experimental models, which have recently been demonstrated to be regulated to some extent, via the role of TAZ and β-catenin by inhibiting their binding Β-TRCP, which increases their stability [88].

In addition, LATS2 is downregulated in 60% of NSCLC cancers, and its high levels led to an improved prognosis and negative regulation of carcinogenic YAP in NSCLC [91]. In addition, MST1 kinase has been demonstrated to inhibit the growth of NSCLC in vitro and in vivo [92]. Notably, Ras-association domain family 1 isoform A (RASSF1A) activates MST1/2 and LATS1 in the presence of DNA damage or other stress signals in NSCLC [93].

#### Breast cancer

An abnormal Hippo pathway facilitates breast cancer metastasis through different mechanisms [94]. The Hippo core components such as YAP, TAZ and MST1, serve a critical role in invasive breast tumor colonization in or outside breast tissue [95]. Relevant data from additional studies demonstrated that the deficiency of YAP decreases the incidence of lung metastasis in a genetically engineered breast cancer mouse model. The presence of Ski effectively suppresses lung metastasis in breast cells following the overexpression of TAZ [96, 97]. The Hippo pathway is also necessary for breast cancer, which can occur in up to 70% of patients with advanced breast cancer. Phosphorylated HER3 Tyr1307 is able to induce MST1 methylation at the lys59 site, thereby producing active YAP/TAZ in tumor cells, thereby promoting metastasis in bone cancer [98]. Bartucci et al. observed that the nuclear expression of TAZ in bone metastasis was significantly higher than its cytoplasmic expression in primary tumors. In addition, a hypoxic microenvironment in bone marrow is also an important cause of tumor infiltration. The hypoxic state is characterized by the presence of hypoxic-inducing factor (HIF)-1α. Bendinelli et al. reported that HIF-1α is able to stimulate bone metastasis in breast cancer by interacting with TAZ in a hypoxic microenvironment [99, 100]. Nuclear HIF-1α has been identified to be largely associated with EMT in breast cancer metastasis, and can also be regulated by the interaction between E-cadherin and Hippo pathway factors [101]. Of note, MST1/2 and LATS1/2, the upstream kinases of the Hippo pathway, can regulate YAP phosphorylation as tumor suppressors. Therefore, LATS1/2 may be a novel target for anticancer treatment in breast cancer [94].

#### Gastric cancer

The high expression of YAP1 in the cytoplasm and nucleus has been observed in dysplasia, gastric adenocarcinoma and metastatic stomach disease [102]. The activation of YAP1 is associated with the poor outcome of early stage GC. As YAP1 has an upward adjustment ability in GC, siRNA-mediated YAP1 knockdown has exhibited inhibitory phonological types, including reduced cell proliferation, inhibition of single-layer colony formation associated with anchorage, and the reduction of cell invasion and migration. In MKN45 cells with negative YAP1 expression, the ectopic expression of YAP1 promotes anchorage dependence or independent colony formation. The ascension of YAP1 in MKN45 cells has also led to more invasive phenotypic changes that can promote the proliferation of cells in vitro and in vivo [103]. YAP1 was further demonstrated to enhance the expression of C-fos induced by serum/EGF in GC cells [104]. The expression of YAP1 in GC was positively correlated with survival [105]. The interaction of YAP1 and RUNX2 increases oncogenic transformation by repression of p21 protein expression [106]. In gastric and lung adenocarcinoma, tyrosine kinase AXL is the direct functional target of YAP1 [107]. Several study groups also provided a comprehensive account of the similar carcinogenic effects of YAP1 in the occurrence and metastasis of GC [108].

TAZ, another key effector of the Hippo pathway, is associated with the abnormal excessive expression of β-catenin, which is associated with a poor prognosis in patients with esophageal and gastric junction adenocarcinoma. The YAP1/TAZ hyperactivation appears to drive tumorigenesis in gastrointestinal cancers, and take over carcinogenesis in a RAS-independent manner [109].

#### Hepatocellular cancer

The overexpression of MST1/2 inhibits cell proliferation and the mRNA expression of CTGF, AREG and Survivin, and promotes YAP1 phosphorylation in the development of HCC [110]. Silencing YAP1 can restore hepatocyte differentiation by siRNA-lipid nanoparticles (siRNA-LNPs) in advanced HCC, and leads to tumor regression [111]. The activation of YAP1 is an early event of HCC and is an independent prognostic factor [112]. The PDZ binding motif in YAP1 is crucial for activating the cell proliferation gene CTGF, which is also a TEAD-dependent transcription target [113]. CREB (cyclic adenosine monophosphate response element-binding protein) promotes the transcriptional output of YAP1 via binding to -608/-439 sites, a novel region of the YAP promoter [114]. The interaction of MEK1-YAP1 is important in cell proliferation and the maintenance of transformed phenotype in HCC cells [115]. AMOT-P130 was associated with YAP1-TEAD transcription complexes, and helped regulate a certain YAP1 targeted genes, which are associated with the occurrence of liver tumors [116]. It has been reported that SIRT1 reacts with the YAP1 protein in HCC cells, thus activating YAPTU/TEAD4 transcription ability and promoting HCC tumor cell growth [117]. In conclusion, YAP1 up-regulates Jag-1 in order to activate Notch signaling in HCC cells, which indicates that a correlation exists between the Hippo pathway and the carcinogenic pathway.

#### Renal cell carcinoma cancer

It has been detected abnormal Hippo pathway signaling in various human cancers can result in hyperactive YAP function, including kidney [118]. The nuclear overexpression of YAP has also been observed in a subset of patients with clear cell Renal Cell Carcinoma (ccRCC) [119]. In ccRCC tissues and cell lines, YAP mRNA and protein expression levels were also observed to be increased. In addition, the knockdown of YAP in the 786-0 ccRCC system causes cells to stop progressing through the cell cycle and an increase in apoptosis [120]. The deletion of the Nf2 gene, which can encode the upstream Hippo pathway regulator Merlin can result in intratubular neoplasia that progresses to invasive carcinoma [121]. Of note, in this model, early lumen-filling lesions exhibited hyperactive EGFR signaling with EGFR inhibition halting tumor cell proliferation. These findings highlight the potentially dangerous effects of uncontrolled YAP signals and further confirm the cross-dialogue between the Hippo and EGFR pathways [122].

#### Colorectal cancer

In CRC, the study of Liang et al. reported a decrease in the mRNA ratio of LATS1 and MST1/2, and an increase in mRNA levels in YAP, TAZ, TEAD and OCT4 when compared with healthy colon samples [123]. The majority of genes that encode Hippo pathway proteins have been demonstrated to exert functions as tumor suppressor or oncogenes in numerous types of cancer. Wang et al. demonstrated that there was a relationship between the expression levels of YAP1 and TAZ and lymph node status in CRC [124]. Other reports have stated the prognostic value of the transcriptional levels of YAP1 and TAZ for patients with CRC. In 522 cases of CRC the mRNA expression rates of TAZ and YAP1 mRNA were positively correlated with their downstream targeted genes AXL and CTGF [125]. In a quantitative PCR study based on the expression of all Hippo pathway elements in CRC, the mRNA levels of MST1 and LATS2 in CRC tissues decreased more than those in colorectal cancer adenomas or adjacent non-tumor tissues [126]. The increase of YAP was also observed in human CRC liver metastases, and was correlated with CRC relapse [126].

### Hippo pathway in leukemia

#### YAP acts as an oncoprotein

YAP can interact with TEAD and form a protein complex termed an oncoprotein, which contributed to the transcription of target downstream genes, including c-Myc and Survivin [127]. It has recently been revealed that porphyrin family members including VP, hematoporphyrin, and protoporphyrin IX can delete the interactions between YAP and TEAD [128]. In addition, subsequent research was performed in chronic myeloid leukemia (CML) samples in order to analyze the expression of MST1/2 and YAP1. The results revealed that the inhibition of YAP results in a marked anti-tumor effect in CML [129], and the inhibitory effect of shRNA and VP on YAP function in HL-60 cells was also reported. Also, the study clearly revealed that silencing of YAP may serve as a potential treatment strategy for acute pro-myelocytic leukemia (APL) [130]. However, further investigations are required in order to assess its suitability in a clinical setting.

#### YAP acts as an anti-cancer protein

Recent data has indicated that YAP1 is stably up-regulated in epithelial tumor cell lines; however, the expression ofYAP1 is markedly reduced in malignant hematologic tumors, including lymphoma, leukemia and multiple myeloma (MM) [131]. Other reports have demonstrated that YAP1 is located in chromosome 11 at site 11q22.1, and there is a missing of the focal homozygous of the site in 5–13% of MM samples [132]. BIRC2 and BIRC3, are the primary targets of this deletion, and have been reported to control the pro-oncogenic NF-κB pathway [133]. In addition, Cottini et al. reported that YAP1was deleted in all examined MM cell lines and the majority of MM samples. Importantly, the survival rate of low expression of YAP1 in MM samples was significantly lower than that of higher expression rate. Additionally, various datasets have reported that the knockdown in YAP1 expression levels can progress from normal plasma cells to MM [131].

### Hippo pathway in sarcoma

#### Osteosarcoma

In osteosarcoma (OS), a report of Chai et al. showed TEAD1 was the major transcription factor of Hippo signaling pathway [134]. Knockdown of TEAD1 suppressed multiple malignant phenotypes of OS, such as cell proliferation, apoptosis resistance and invasive potential through regulation of PTGS2 and CRY61 [134]. YAP1 is highly expressed and predicts a poor prognosis. Silencing of YAP1 may inhibit OS cells growth and metastasis [135]. Besides YAP1, TAZ has also been reported be responsible for the progression of OS [136].

#### Rhabdomyosarcoma

YAP has recently been identified as a potent driver of embryonal rhabdomyosarcoma (ERMS). Constitutive YAP hyperexpression in activated muscle stem cells causes ERMS-like tumors [137]. In contrast to YAP, TAZ was reported to promote myogenic differentiation, which would be anti-tumorigenic [138]. However, Mohamed et al. demonstrated TAZ functions as an oncogene in ERMS. TAZ drives expression of ERMS stem cell factor Myf5, and promote proliferation of ERMS cells [139]. Therefore, more researches need to carry out to confirm whether TAZ is an oncogene or suppressor in ERMS.

#### Angiosarcoma

Human angiosarcoma is a rare malignant vascular tumor associated with extremely poor clinical outcome and generally arising in skin of the head and neck region. Recent research demonstrated YAP is specifically expressed and translocated in the nucleus in angiosarcomas; and is a key modulator of proliferation in human angiosarcomas and inhibition of survivin activity may be a potential therapeutic target [140].

### Current drugs targeting the hippo pathway for cancer treatment

**Fig. 6:**
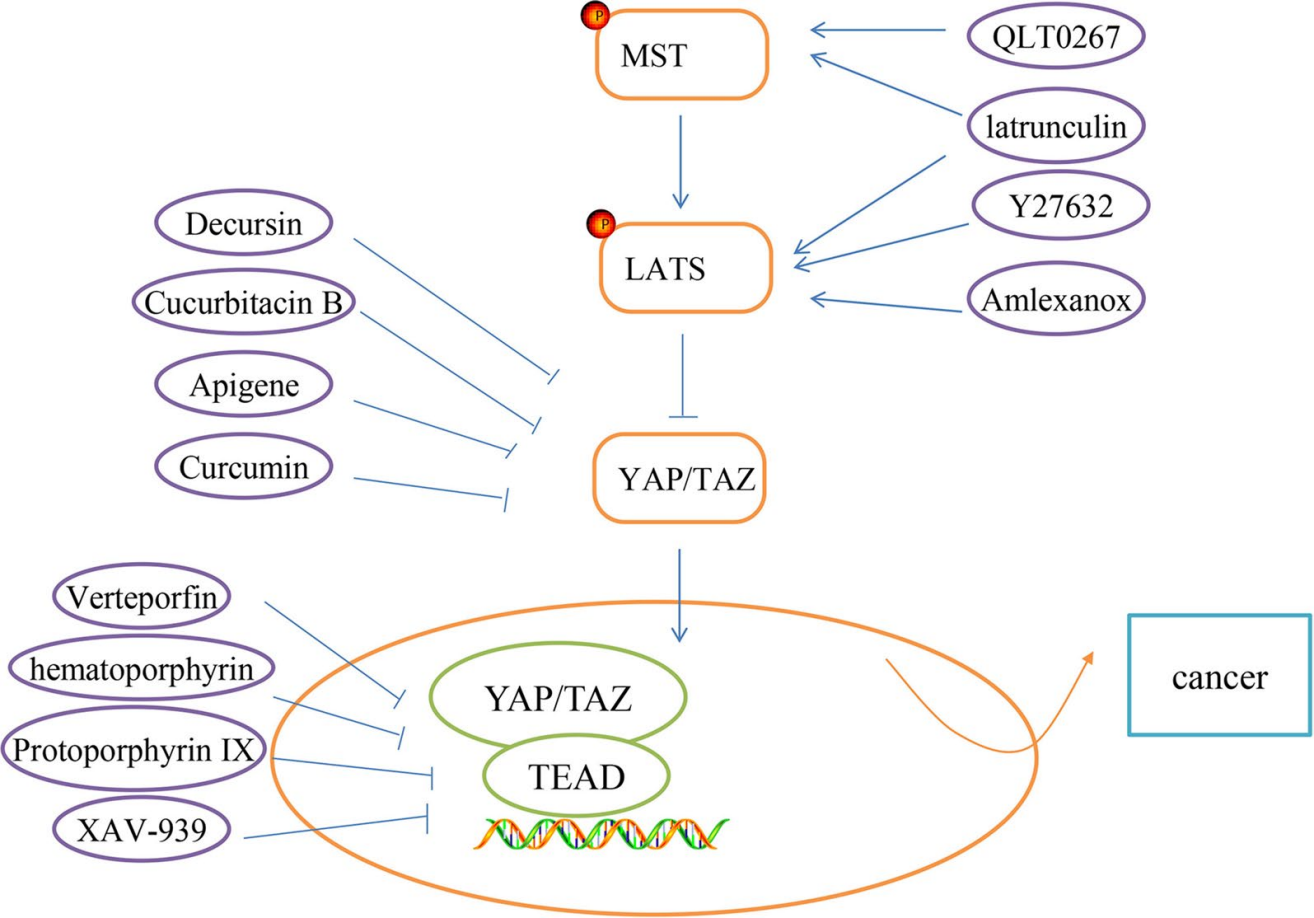
Hippo pathway-targeted inhibitors. Current Hippo pathway-targeted drugs have been discussed in the review, as shown in the figure

With the ever-greater understanding of cellular signaling mechanisms and genetic alterations in carcinogenesis, considerable progress in cancer treatment has been made in recent years. A great number of targeted drugs has been identified to improve survival rate of cancer patients, such as ibrutinib (BTK inhibitor), idelaisib (PI3Kδ inhibitor), and ciclesonide (Smo inhibitor). As we mentioned before, hippo pathway is a major signaling pathway in the human body, and is responsible for cancer development. Therefore, it is important to study hippo pathway-targeted drugs to promote cancer-treatment methods. In our review, we summarized several hippo pathway-targeted drugs (Fig. 6).

### MST and LATS activation

F-actin is the inhibitor of MST/LATS, thereby negative regulators of F-actin can indirectly activate MST/LATS activity. The marine-derived macrolides latrunculin are known to disrupt F-actin polymerization. Konishi et al. reported latrunculinA has a strong anti-cancer effect in a peritoneal dissemination model of human gastric cancer in mice [141]. Besides, Y27632 could activate MST/LATS via inhibiting Rho-associated, coiled–coil containing protein kinase (ROCK). Leonel et al. demonstrated Y27632 can inhibit Epithelial-mesenchymal Transition (EMT) of breast cancer cell lines [142].

Additionally, a new study showed that IKBKE directly targets LATS1/2 and induces degradation of LATS1/2, thereby inhibiting the activity of the Hippo pathway [143]. Amlexanox, a selective inhibitor of IKBKE, could be a potential LATS1/2-target drug [143]. ILK (integrin-linked kinase) is a critical negative regulator of the Hippo tumor suppressor pathway, which can prevent Merlin dephosphorylation and activation by suppressing MYPT1, resulting in the nuclear accumulation of YAP/TAZ [144]. QLT0267, an inhibitor of ILK, reduces breast cancer cell growth by activating MST [144].

### Targeting YAP/TAZ regulators

Decursin is a major compound isolated from the root of the Korean Dang-Gui. Recent research indicated that Decursin shows anti-cancer therapy sensitization. Li et al. reported Decursin enables inhibition of YAP activities that functions by the upregulation of LATS1 phosphorylation and ubiquitin E3 ligase, βTRCP in HepG2 hepatocellular carcinoma cells [145]. Cucurbitacins are natural tetracyclic triterpene compounds extracted from several plant families such as Cruciferae and Cucurbitaceae. These compounds have a broad spectrum of pharmacological activities including antioxidant, anticancer, and antidiabetic. Chai et al. reported Cucurbitacin B exerts anticancer activities in colorectal cancer cells by inhibiting YAP protein [146].

Apigenin is a widely distributed flavonoid in vegetables and fruits. Recent studies have showed that Apigenin has anti-cancer activity. Li et al. indicated that Apigenin decreased YAP/TAZ activity and the expression of target genes, such as CTGF and CYR61 in TNBC cells. Apigenin is a promising therapeutic agent for the treatment of TNBC patients by inhibiting YAP/TAZ activity [147]. Curcumin, a hydrophobic polyphenol derived from turmeric (Curcuma Longa), is one of the most-studied plant-derived natural products. Curcumin has shown anti-proliferation, apoptosis induction and anti-invasion in various human cancers. Gao et al. demonstrated Curcumin could down-regulate the expression of YAP/TAZ to suppress bladder cancer development, which suggested the therapeutic potential of Curcumin in the treatment of bladder cancer [148].

### Inhibition of YAP/TAZ-TEAD interaction

According to preceding investigation, Verteporfin (VP), a YAP specific inhibitor, can block the interaction between YAP and TEAD to repress YAP’s function [149]. Several researchers discovered the VP is able to restrain cancer cell growth in some tumors, such as retinoblastoma, endometrial and ovarian cancers. Besides VP, the other members of the porphyrin family, such as hematoporphyrin and protoporphyrin IX are both currently identified as disruptors of YAP-TEAD interaction in xenograft mouse models [150], which could be the next candidates for cancer treatment. In addition, Jia et al. reported XAV-939, a Tankyrase inhibitor, can decrease YAP protein levels and inhibit YAP-TEAD luciferase reporter activity [151].

## Conclusion

Taken together, the Hippo pathway plays an essential role in most of cancers, which makes it to be a critical field for further investigation. From the data described, the hippo pathway is the opportunities and challenges to the treatment of cancer. A large number of solid cancers showed higher-expression of YAP and TAZ, knockdown or silencing them will be responsible for cancer treatment. However, based on the perspectives by research, YAP can be an oncoprotein or anti-tumor molecular in malignant hematologic tumors. Other core components of Hippo pathway such as LATS1/2 also are critical for cancer therapy as negative effectors. Notably, Hippo pathway is regulated by a complex network of signaling pathway, and microRNAs, circRNAs, as well as zinc-finger proteins. Hippo pathway-targeted drugs have been identified to treat cancer. Despite tremendous researches on Hippo pathway have been reported, further knowledge upon it remains unknown and needs to be exported.

## References

[1] Zhu C, Li L, Zhao B (2015). The regulation and function of YAP transcription co-activator. Acta Biochim Biophys Sin (Shanghai).

[2] Misra JR, Irvine KD (2018). The Hippo signaling network and its biological functions. Ann Rev Genet.

[3] Praskova M, Xia F, Avruch J (2008). MOBKL1A/MOBKL1B phosphorylation by MST1 and MST2 inhibits cell proliferation. Curr Biol.

[4] Bae SJ, Ni L, Osinski A, Tomchick DR, Brautigam CA, Luo X (2017). SAV1 promotes Hippo kinase activation through antagonizing the PP2A phosphatase STRIPAK. Elife..

[5] Zanconato F, Battilana G, Cordenonsi M, Piccolo S (2016). YAP/TAZ as therapeutic targets in cancer. Curr Opin Pharmacol.

[6] Zanconato F, Cordenonsi M, Piccolo S (2016). YAP/TAZ at the roots of cancer. Cancer Cell.

[7] Dong J, Feldmann G, Huang J, Wu S, Zhang N, Comerford SA (2007). Elucidation of a universal size-control mechanism in Drosophila and mammals. Cell.

[8] Zanconato F, Forcato M, Battilana G, Azzolin L, Quaranta E, Bodega B (2015). Genome-wide association between YAP/TAZ/TEAD and AP-1 at enhancers drives oncogenic growth. Nat Cell Biol.

[9] Macias MJ, Hyvonen M, Baraldi E, Schultz J, Sudol M, Saraste M (1996). Structure of the WW domain of a kinase-associated protein complexed with a proline-rich peptide. Nature.

[10] Yagi R, Chen LF, Shigesada K, Murakami Y, Ito Y (1999). A WW domaincontaining Yes-associated protein (YAP) is a novel transcriptional co-activator. EMBO J.

[11] Oka T, Remue E, Meerschaert K, Vanloo B, Boucherie C, Gfeller D (2010). Functional complexes between YAP2 and ZO-2 are PDZ domain-dependent, and regulate YAP2 nuclear localization and signalling. Biochem J..

[12] Kowalik MA, Saliba C, Pibiri M, Perra A, Ledda-Columbano GM, Sarotto I (2011). Yes-associated protein regulation of adaptive liver enlargement and hepatocellular carcinoma development in mice. Hepatology.

[13] Moon S, Kim W, Kim S, Kim Y, Song Y, Bilousov O (2017). Phosphorylation by NLK inhibits YAP-14-3-3- interactions and induces its nuclear localization. EMBO Rep.

[14] Liu CY, Zha ZY, Zhou X, Zhang H, Huang W, Zhao D (2010). The Hippo tumor pathway promotes TAZ degradation by phosphorylating a phosphodegron and recruiting the SCFβ-TrCP E3 ligase. J Biol Chem.

[15] Varelas X (2014). The Hippo pathway effectors TAZ and YAP in development, homeostasis and disease. Development..

[16] Hansen CG, Moroishi T, Guan KL (2015). YAP and TAZ: a nexus for Hippo signaling and beyond. Trends Cell Biol.

[17] Salah Z, Alian A, Aqeilan RI (2012). WW domain-containing proteins: retrospectives and the future. Front Biosci.

[18] Ye F, Zhang M (2013). Structures and target recognition modes of PDZ domains: recurring themes and emerging pictures. Biochem J..

[19] Chen L, Chan SW, Zhang X, Walsh M, Lim CJ, Hong W (2010). Structural basis of YAP recognition by TEAD4 in the hippo pathway. Genes Dev.

[20] Howell M, Borchers C, Milgram SL (2004). Heterogeneous nuclear ribonuclear protein U associates with YAP and regulates its co-activation of Bax transcription. J Biol Chem.

[21] Sudol M (1994). Yes-associated protein (YAP65) is a proline-rich phosphoprotein that binds to the SH3 domain of the Yes proto-oncogene product. Oncogene.

[22] Nam HK, Yoonmi L, Jong IY (2018). Dishevelling Wnt and Hippo. BMB Rep..

[23] Hyun WP, Young CK, Bo Y, Toshiro M, Jung-Soon M, Steven WP (2015). Alternative Wnt signaling activates YAP/TAZ. Cell.

[24] Mo JS, Meng Z, Kim YC, Park HW, Hansen CG, Kim S (2015). Cellular energy stress induces AMPK-mediated regulation of YAP and the Hippo pathway. Nat Cell Biol.

[25] DeRan M, Yang J, Shen CH, Peters EC, Fitamant J, Chan P (2014). Energy stress regulates hippo-YAP signaling involving AMPK-mediated regulation of angiomotin-like 1 protein. Cell Rep..

[26] Gailite I, Aerne BL, Tapon N (2015). Differential control of Yorkie activity by LKB1/AMPK and the Hippo/Warts cascade in the central nervous system. Proc Natl Acad Sci.

[27] Ja HK, Kun LG (2018). Interplay between YAP/TAZ and metabolism. Cell Metab..

[28] Saito A, Suzuki HI, Horie M, Ohshima M, Morishita Y, Abiko Y (2013). An integrated expression profiling reveals target genes of TGF-β and TNF-β possibly mediated by microRNAs in lung cancer cells. PLoS ONE.

[29] Akira S, Takahide N (2015). Hippo and TGF-βinterplay in the lung field. Am J Physiol Lung Cell Mol Physiol.

[30] Varelas X, Sakuma R, Samavarchi-Tehrani P, Peerani R, Rao BM, Dembowy J (2008). TAZ controls Smad nucleocytoplasmic shuttling and regulates human embryonic stem-cell self-renewal. Nat Cell Biol.

[31] Beyer TA, Weiss A, Khomchuk Y, Huang K, Ogunjimi AA, Varelas X (2013). Switch enhancers interpret TGF-β and Hippo signaling to control cell fate in human embryonic stem cells. Cell Rep..

[32] Shen Z, Stanger BZ (2015). YAP regulates S-phase entry in endothelial cells. PLoS ONE.

[33] Benham-Pyle BW, Pruitt BL, Nelson WJ (2015). Cell adhesion. Mechanical strain induces E-cadherin-dependent Yap1 and beta-catenin activation to drive cell cycle entry. Science..

[34] Ruth N, Chung JT, Hyunbum J, Tamás K, Peter C (2016). Oncogenic KRAS signaling and YAP1/β-catenin: similar cell cycle control in tumor initiation. Semin Cell Dev Biol.

[35] Kapoor A, Yao W, Ying H, Hua S, Liewen A, Wang Q (2014). Yap1 activation enables bypass of oncogenic Kras addiction in pancreatic cancer. Cell.

[36] Wei GH, Badis G, Berger MF, Kivioja T, Palin K, Enge M (2010). Genome-wide analysis of ETS-family DNA-binding in vitro and in vivo. EMBO J.

[37] Nguyen LT, Tretiakova MS, Silvis MR, Lucas J, Klezovitch O, Coleman I (2015). ERG activates the YAP1 transcriptional program and induces the development of age-related prostate tumors. Cancer Cell.

[38] Yulian M, Victor D, Michał AS, Yuriy B, Yuriy D, Askold K (2018). Potential clinical applications of microRNAs as biomarkers for renal cell carcinoma. Cent Eur J Urol..

[39] Goh JN, Loo SY, Datta A, Siveen KS, Yap WN, Cai W (2016). microRNAs in breast cancer: regulatory roles governing the hallmarks of cancer. Biol Rev Camb Philos Soc.

[40] Hawkes JE, Nguyen GH, Fujita M, Florell SR, Callis Duffin K, Krueger GG (2016). microRNAs in psoriasis. J Invest Dermatol..

[41] Hai NZ, Qiao QX, Abhimanyu T, Martin OA, Manas C, Arunima G (2018). Endothelial dysfunction in diabetes and hypertension: role of microRNAs and long non-coding RNAs. Life Sci.

[42] Hanif Q, Farooq M, Amin I, Mansoor S, Zhang Y, Khan QM (2018). In silico identification of conserved miRNAs and their selective target gene prediction in indicine (*Bos indicus*) cattle. PLoS ONE.

[43] Xu X, Tao Y, Shan L, Chen R, Jiang H, Qian Z (2018). The role of microRNAs in hepatocellular carcinoma. J Cancer..

[44] Song S, Ajani JA, Honjo S, Maru DM, Chen Q, Scott AW (2014). Hippo coactivator YAP1 upregulates SOX9 and endows esophageal cancer cells with stem-like properties. Cancer Res.

[45] Choe MH, Yoon Y, Kim J, Hwang SG, Han YH, Kim JS (2018). miR-550a-3-5p acts as a tumor suppressor and reverses BRAF inhibitor resistance through the direct targeting of YAP. Cell Death Dis..

[46] Li Z, Wang G, Feng D, Zu G, Li Y, Shi X (2018). Targeting the miR-665-3p-ATG4B-autophagy axis relieves inflammation and apoptosis in intestinal ischemia/reperfusion. Cell Death Dis..

[47] Hu Y, Yang C, Yang S, Cheng F, Rao J, Wang X (2018). miR-665 promotes hepatocellular carcinoma cell migration, invasion, and proliferation by decreasing Hippo signaling through targeting PTPRB. Cell Death Dis..

[48] Luo Q, Wei C, Li X, Li J, Chen L, Huang Y (2014). MicroRNA-195-5p is a potential diagnostic and therapeutic target for breast cancer. Oncol Rep.

[49] Wang F, Jiang C, Sun Q, Yan F, Wang L, Fu Z (2015). miR-195 is a key regulator of Raf1 in thyroid cancer. Oncol Targets Ther..

[50] Yu S, Jing L, Yin XR, Wang MC, Chen YM, Guo Y (2017). MiR-195 suppresses the metastasis and epithelial-mesenchymal transition of hepatocellular carcinoma by inhibiting YAP. Oncotarget..

[51] Perra A, Kowalik MA, Ghiso E, Ledda-Columbano GM, Di Tommaso L, Angioni MM (2014). YAP activation is an early event and a potential therapeutic target in liver cancer development. J Hepatol.

[52] Bera R, Chiou CY, Yu MC, Peng JM, He CR, Hsu CY (2014). Functional genomics identified a novel protein tyrosine phosphatase receptor type F-mediated growth inhibition in hepatocarcinogenesis. Hepatology.

[53] Zhao B, Lei Q, Guan KL (2009). Mst out and HCC. Cancer Cell.

[54] Cheng L, Wang H, Han S (2017). MiR-3910 promotes the growth and migration of cancer cells in the progression of hepatocellular carcinoma. Dig Dis Sci.

[55] Que K, Tong Y, Que G, Li L, Lin H, Huang S (2017). Downregulation of miR-874-3p promotes chemotherapeutic resistance in colorectal cancer via inactivation of the Hippo signaling pathway. Oncol Rep.

[56] Jeck WR, Sharpless NE (2014). Detecting and characterizing circular RNAs. Nat Biotechnol.

[57] Ashwal-Fluss R, Meyer M, Pamudurti NR, Ivanov A, Bartok O, Hanan M (2014). circRNA biogenesis competes with pre-mRNA splicing. Mol Cell.

[58] Lorena V, Maria F, Andrea S, Federica G, Jlenia V, Teresa C (2017). The oncogenic role of circPVT1 in head and neck squamous cell carcinoma is mediated through the mutant p53/YAP/TEAD transcription-competent complex. Genome Biol.

[59] Xiao Z, Yan FX, Zi JQ, Wei SZ, Qi W, Yan C (2018). circRNA_104075 stimulates YAP-dependent tumorigenesis through the regulation of HNF4a and may serve as a diagnostic marker in hepatocellular carcinoma. Cell Death Dis..

[60] Zhang J, Zhao X, Zhang J, Zheng X, Li F (2018). Circular RNA hsa_circ_0023404 exerts an oncogenic role in cervical cancer through regulatingmiR-136/TFCP2/YAP pathway. Biochem Biophys Res Commun.

[61] Memczak S, Jens M, Elefsinioti A, Torti F, Krueger J, Rybak A (2013). Circular RNAs are a large class of animal RNAs with regulatory potency. Nature.

[62] Chen J, Li Y, Zheng Q, Bao C, He J, Chen B (2017). Circular RNA profile identifies circPVT1 as a proliferative factor and prognostic marker in gastric cancer. Cancer Lett.

[63] Panda AC, Grammatikakis I, Kim KM, De S, Martindale JL, Munk R (2017). Identification of senescence-associated circular RNAs (SAC-RNAs) reveals senescence suppressor CircPVT1. Nucleic Acids Res.

[64] Colombo T, Farina L, Macino G, Paci P (2015). PVT1: a rising star among oncogenic long noncoding RNAs. Biomed Res Int.

[65] Lorena V, Maria F, Andrea S, Federica G, Jlenia V, Teresa C (2017). The oncogenic role of circPVT1 in head and neck squamous cell carcinoma is mediated through the mutant p53/YAP/TEAD transcription-competent complex. Genome Biol.

[66] Seetharaman S, Flemyng E, Shen J, Conte MR, Ridley AJ (2016). The RNA-binding protein LARP4 regulates cancer cell migration and invasion. Cytoskeleton (Hoboken)..

[67] Zhang J, Liu H, Hou L, Wang G, Zhang R, Huang Y (2017). Circular RNA_LARP4 inhibits cell proliferation and invasion of gastric cancer by sponging miR-424-5p and regulating LATS1 expression. Mol Cancer..

[68] Cassandri M, Smirnov A, Novelli F, Pitolli C, Agostini M, Malewicz M (2017). Zinc-finger proteins in health and disease. Cell Death Discov..

[69] Jen J, Wang YC (2016). Zinc finger proteins in cancer progression. J Biomed Sci..

[70] Andreini C, Bertini I, Cavallaro G (2011). Minimal functional sites allow a classification of zinc sites in proteins. PLoS ONE.

[71] Eom KS, Cheong JS, Lee SJ (2016). Structural analyses of zinc finger domains for specific interactions with DNA. J Microbiol Biotechnol..

[72] Krishna SS, Majumdar I, Grishin NV (2003). Structural classification of zinc fingers: survey and summary. Nucleic Acids Res.

[73] Hossain MA, Barrow JJ, Shen Y, Haq MI, Bungert J (2015). Artificial zinc finger DNA binding domains: versatile tools for genome engineering and modulation of gene expression. J Cell Biochem.

[74] Gray KA, Yates B, Seal RL, Wright MW, Bruford EA (2015). Genenames.org: the HGNC resources in 2015. Nucleic Acids Res..

[75] Uhlen M, Fagerberg L, Hallstrom BM, Lindskog C, Oksvold P, Mardinoglu A (2015). Proteomics: tissue-based map of the human proteome. Science.

[76] Eguchi T, Prince T, Wegiel B (2015). Role and regulation of myeloid zinc finger protein 1 in cancer. J Cell Biochem.

[77] Verma NK, Gadi A, Maurizi G, Roy UB, Mansukhani A, Basilico C (2017). Myeloid zinc finger 1 and GA binding protein co-operate with Sox2 in regulating the expression of yes-associated protein 1 in cancer cells. Stem Cells..

[78] Schelleckes K, Schmitz B, Lenders M, Mewes M, Brand SM, Brand E (2018). ZFP226 is a novel artificial transcription factor for selective activation of tumor suppressor KIBRA. Sci Rep..

[79] Guske K (2014). Tissue-specific differences in the regulation of KIBRA gene expression involve transcription factor TCF7L2 and a complex alternative promoter system. J Mol Med (Berl)..

[80] Yu FX, Guan KL (2013). The Hippo pathway, regulators and regulations. Genes..

[81] Thiery JP, Acloque H, Huang RY, Nieto MA (2009). Epithelial-mesenchymal transitions in development and disease. Cell.

[82] Vandewalle C, Van Roy F, Berx G (2009). The role of the ZEB family of transcription factors in development and disease. Cell Mol Life Sci.

[83] Lehmann W, Mossmann D, Kleemann J, Mock K, Meisinger C, Brummer T (2016). ZEB1 turns into a transcriptional activator by interacting with YAP1 in aggressive cancer types. Nat Commun..

[84] Lei L, Wu J, Gu D, Liu H, Wang S (2016). CIZ1 interacts with YAP and activates its transcriptional activity in hepatocellular carcinoma cells. Tumour Biol.

[85] Chen HY, Yu SL, Ho BC, Su KY, Hsu YC, Chang CS (2015). R331W missense mutation of oncogene YAP1 is a germline risk allele for lung adenocarcinoma with medical actionability. J Clin Oncol.

[86] Lau AN, Curtis SJ, Fillmore CM, Rowbotham SP, Mohseni M, Wagner DE (2014). Tumor-propagating cells and YAP/TAZ activity contribute to lung tumor progression and metastasis. EMBO J.

[87] Zhang W, Gao Y, Li F, Tong X, Ren Y, Han X (2015). YAP promotes malignant progression of LKB1-deficient lung adenocarcinoma through downstream regulation of survivin. Cancer Res.

[88] Lo Sardo F, Strano S, Blandino G (2018). YAP and TAZ in lung cancer: oncogenic role and clinical targeting. Cancers (Basel)..

[89] Testoni E, Stephenson NL, Torres-Ayuso P, Marusiak AA, Trotter EW, Hudson A (2016). Somatically mutated ABL1 is an actionable and essential NSCLC survival gene. EMBO Mol Med.

[90] Yang CH, Chou HC, Fu YN, Yeh CL, Cheng HW, Chang IC (2015). EGFR over-expression in non-small cell lung cancers harboring EGFR mutations is associated with marked down-regulation of CD82. Biochim Biophys Acta.

[91] Malik SA, Khan MS, Dar M, Hussain MU, Shah MA, Shafi SM (2018). Molecular alterations and expression dynamics of LATS1 and LATS2 genes in non-small-cell lung carcinoma. Pathol Oncol Res..

[92] Xu CM, Liu WW, Liu CJ, Wen C, Lu HF, Wan FS (2013). MST1 overexpression inhibited the growth of human non-small cell lung cancer in vitro and in vivo. Cancer Gene Ther.

[93] Agathanggelou A, Honorio S, Macartney DP, Martinez A, Dallol A, Rader J (2001). Methylation associated inactivation of RASSF1A from region 3p213 in lung, breast and ovarian tumours. Oncogene..

[94] Wei C, Wang Y, Li X (2018). The role of Hippo signal pathway in breast cancer metastasis. Onco Targets Ther..

[95] Bos PD, Zhang XHF, Nadal C, Shu W, Gomis RR, Nguyen DX (2009). Genes that mediate breast cancer metastasis to the brain. Nature.

[96] Chen Q, Zhang N, Gray RS, Li H, Ewald AJ, Zahnow CA (2014). A temporal requirement for Hippo signalling in mammary gland differentiation, growth and tumorigenesis. Genes Dev.

[97] Rashidian J, Le Scolan E, Ji X, Zhu Q, Mulvihill MM, Nomura D (2015). Ski regulates Hippo and TAZ signalling to suppress breast cancer progression. Sci Signal..

[98] Li C, Wang S, Xing Z (2017). A ROR1-HER3-lncRNA signalling axis modulates the Hippo-YAP pathway to regulate bone metastasis. Nat Cell Biol.

[99] Xiang L, Gilkes DM, Hu H, Luo W, Bullen JW, Liang H (2015). HIF-1α and TAZ serve as reciprocal co-activators in human breast cancer cells. Oncotarget..

[100] Bendinelli P, Maroni P, Matteucci E, Luzzati A, Perrucchini G, Desiderio MA (2013). Hypoxia inducible factor-1is activated by transcriptional co-activator with PDZ-binding motif (TAZ) versus WW domain-containing oxidoreductase (WWOX) in hypoxic microenvironment of bone metastasis from breast cancer. Eur J Cancer.

[101] Maroni P, Matteucci E, Drago L, Banfi G, Bendinelli P, Desiderio MA (2015). Hypoxia induced E-cadherin involving regulators of Hippo pathway due to HIF-1 stabilization/nuclear translocation in bone metastasis from breast carcinoma. Exp Cell Res.

[102] Lam-Himlin DM, Daniels JA, Gayyed MF, Dong J, Maitra A, Pan D (2006). The hippo pathway in human upper gastrointestinal dysplasia and carcinoma: a novel oncogenic pathway. Int J Gastrointest Cancer..

[103] Wei K, Alfred SC, Jun Y, Ka FT (2016). Emerging role of Hippo pathway in gastric and other gastrointestinal cancers. World J Gastroenterol.

[104] Kang W, Tong JH, Chan AW, Lee TL, Lung RW, Leung PP (2011). Yes-associated protein1 exhibits oncogenic property in gastric cancer and its nuclear accumulation associates with poor prognosis. Clin Cancer Res.

[105] Da CL, Xin Y, Zhao J, Luo XD (2009). Significance and relationship between Yes-associated protein and survivin expression in gastric carcinoma and precancerous lesions. World J Gastroenterol.

[106] Vitolo MI, Anglin IE, Mahoney WM, Renoud KJ, Gartenhaus RB, Bachman KE (2007). The RUNX2 transcription factor cooperates with the YES-associated protein, YAP65, to promote cell transformation. Cancer Biol Ther.

[107] Cui ZL, Han FF, Peng XH, Chen X, Luan CY, Han RC (2012). YES-associated protein 1 promotes adenocarcinoma growth and metastasis through activation of the receptor tyrosine kinase Axl. Int J Immunopathol Pharmacol..

[108] Hu X, Xin Y, Xiao Y, Zhao J (2014). Overexpression of YAP1 is correlated with progression, metastasis and poor prognosis in patients with gastric carcinoma. Pathol Oncol Res..

[109] Sun L, Chen F, Shi W, Qi L, Zhao Z, Zhang J (2014). Prognostic impact of TAZ and β-catenin expression in adenocarcinoma of the esophagogastric junction. Diagn Pathol..

[110] Wang C, Zhu ZM, Liu CL, He XJ, Zhang HY, Dong JH (2015). Knockdown of yes-associated protein inhibits proliferation and downregulates large tumor suppressor 1 expression in MHCC97H human hepatocellular carcinoma cells. Mol Med Rep..

[111] Fitamant J, Kottakis F, Benhamouche S, Tian HS, Chuvin N, Parachoniak CA (2015). YAP inhibition restores hepatocyte differentiation in advanced HCC, leading to tumor regression. Cell Rep..

[112] Han SX, Bai E, Jin GH, He CC, Guo XJ, Wang LJ (2014). Expression and clinical significance of YAP, TAZ, and AREG in hepatocellular carcinoma. J Immunol Res..

[113] Shimomura T, Miyamura N, Hata S, Miura R, Hirayama J, Nishina H (2014). The PDZ-binding motif of Yes-associated protein is required for its co-activation of TEAD-mediated CTGF transcription and oncogenic cell transforming activity. Biochem Biophys Res Commun.

[114] Wang J, Ma L, Weng W, Qiao Y, Zhang Y, He J (2013). Mutual interaction between YAP and CREB promotes tumorigenesis in liver cancer. Hepatology.

[115] Li L, Wang J, Zhang Y, Zhang Y, Ma L, Weng W (2013). MEK1 promotes YAP and their interaction is critical for tumorigenesis in liver cancer. FEBS Lett.

[116] Yi C, Shen Z, Stemmer-Rachamimov A, Dawany N, Troutman S, Showe LC (2013). The p130 isoform of angiomotin is required for Yap-mediated hepatic epithelial cell proliferation and tumorigenesis. Sci Signal..

[117] Mao B, Hu F, Cheng J, Wang P, Xu M, Yuan F (2014). SIRT1 regulates YAP2-mediated cell proliferation and chemoresistance in hepatocellular carcinoma. Oncogene.

[118] Philips GK, Atkins MB (2014). New agents and new targets for renal cell carcinoma. Am Soc Clin Oncol Educ Book..

[119] Schutte U, Bisht S, Heukamp LC, Kebschull M, Florin A, Haarmann J (2014). Hippo signaling mediates proliferation, invasiveness, and metastatic potential of clear cell renal cell carcinoma. Transl Oncol..

[120] Cao JJ, Zhao XM, Wang DL, Chen KH, Sheng X, Li WB (2014). YAP is overexpressed in clear cell renal cell carcinoma and its knockdown reduces cell proliferation and induces cell cycle arrest and apoptosis. Oncol Rep.

[121] Morris ZS, McClatchey AI (2009). Aberrant epithelial morphology and persistent epidermal growth factor receptor signaling in a mouse model of renal carcinoma. Proc Natl Acad Sci.

[122] Wong JS, Meliambro K, Ray J, Campbell KN (2016). Hippo signaling in the kidney: the good and the bad. Am J Physiol Renal Physiol.

[123] Liang K, Zhou G, Zhang Q, Li J, Zhang C (2014). Expression of hippo pathway in colorectal cancer. Saudi J Gastroenterol..

[124] Wang L, Shi S, Guo Z, Zhang X, Han S, Yang A (2013). Overexpression of YAP and TAZ is an independent predictor of prognosis in colorectal cancer and related to the proliferation and metastasis of colon cancer cells. PLoS ONE..

[125] Yuen HF, McCrudden CM, Huang YH, Tham JM, Zhang X, Zeng Q (2013). TAZ expression as a prognostic indicator in colorectal cancer. PLoS ONE.

[126] Wierzbicki PM, Rybarczyk A (2015). The Hippo pathway in colorectal cancer. Folia Histochem Cytobiol.

[127] Pei T, Li Y, Wang J, Wang H, Liang Y, Shi H (2015). YAP is a critical oncogene in human cholangiocarcinoma. Oncotarget..

[128] Gibault F, Corvaisier M, Bailly F, Huet G, Melnyk P, Cotelle P (2016). Non-photoinduced biological properties of verteporfin. Curr Med Chem.

[129] Li H, Huang Z, Gao M, Huang N, Luo Z, Shen H (2016). Inhibition of YAP suppresses CML cell proliferation and enhances efficacy of imatinib in vitro and in vivo. J Exp Clin Cancer Res.

[130] Chen M, Wang J, Yao SF, Zhao Y, Liu L, Li LW (2017). Effect of YAP inhibition on human leukemia HL-60 cells. Int J Med Sci..

[131] Cottini F, Hideshima T, Xu C, Sattler M, Dori M, Agnelli L (2014). Rescue of Hippo coactivator YAP1 triggers DNA damage-induced apoptosis in hematological cancers. Nat Med.

[132] Keats JJ, Fonseca R, Chesi M, Schop R, Baker A, Chng WJ (2007). Promiscuous mutations activate the noncanonical NF-κB pathway in multiple myeloma. Cancer Cell.

[133] Walker BA, Leone PE, Chiecchio L, Dickens NJ, Jenner MW, Boyd KD (2010). A compendium of myeloma-associated chromosomal copy number abnormalities and their prognostic value. Blood.

[134] Chai J, Xu S, Guo F (2017). TEAD1 mediates the oncogenic activities of Hippo-YAP1 signaling in osteosarcoma. Biochem Biophys Res Commun.

[135] Basu-Roy U, Han E, Rattanakorn K, Gadi A, Verma N, Maurizi G (2016). PPARγ agonists promote differentiation of cancer stem cells by restraining YAP transcriptionalactivity. Oncotarget..

[136] Ma J, Huang K, Ma Y, Zhou M, Fan S (2017). The TAZ-miR-224-SMAD4 axis promotes tumorigenesis in osteosarcoma. Cell Death Dis..

[137] Tremblay AM, Missiaglia E, Galli GG, Hettmer S, Urcia R, Carrara M (2014). The Hippo transducer YAP1 transforms activated satellite cells and is a potent effector of embryonal rhabdomyosarcoma formation. Cancer Cell.

[138] Keller C, Guttridge DC (2013). Mechanisms of impaired differentiation in rhabdomyosarcoma. FEBS J.

[139] Abdalla M, Congshan S, Vanessa DM, Joanna S, Edoardo M, Janet S (2016). The Hippo effector TAZ (WWTR1) transforms myoblasts and TAZ abundance is associated with reduced survival in embryonal rhabdomyosarcoma. J Pathol.

[140] Masayuki T, Takao K, Taisuke M, Akihiko Y, Kayo K, Aoi O (2017). Survivin: a novel marker and potential therapeutic target for human angiosarcoma. Cancer Sci.

[141] Konishi H, Kikuchi S, Ochiai T, Ikoma H, Kubota T, Ichikawa D (2009). Latrunculin a has a strong anticancer effect in a peritoneal dissemination model of human gastriccancer in mice. Anticancer Res.

[142] Leonel C, Ferreira LC, Borin TF, Moschetta MG, Freitas GS, Haddad MR (2017). Inhibition of epithelial–mesenchymal transition in response to treatment with metformin and Y27632 in breast cancer cell lines. Anticancer Agents Med Chem.

[143] Liu Y, Lu J, Zhang Z, Zhu L, Dong S, Guo G (2017). Amlexanox, a selective inhibitor of IKBKE, generates anti-tumoral effects by disrupting the Hippopathway in human glioblastoma cell lines. Cell Death Dis..

[144] Serrano I, McDonald PC, Lock F, Muller WJ, Dedhar S (2013). Inactivation of the Hippo tumour suppressor pathway by integrin-linked kinase. Nat Commun..

[145] Li J, Wang H, Wang L, Tan R, Zhu M, Zhong X (2018). Decursin inhibits the growth of HepG2 hepatocellular carcinoma cells via Hippo/YAP signalingpathway. Phytother Res..

[146] Chai Y, Xiang K, Wu Y, Zhang T, Liu Y, Liu X (2018). Cucurbitacin B inhibits the Hippo-YAP signaling pathway and exerts anticancer activity in colorectal cancer cells. Med Sci Monit.

[147] Li YW, Xu J, Zhu GY, Huang ZJ, Lu Y, Li XQ (2018). al Apigenin suppresses the stem cell-like properties of triple-negative breast cancer cells by inhibiting YAP/TAZ activity. Cell Death Discov.

[148] Gao Y, Shi Q, Xu S, Du C, Liang L, Wu K (2014). Curcumin promotes KLF5 proteasome degradation through downregulating YAP/TAZ in bladdercancer cells. Int J Mol Sci.

[149] Dong L, Lin F, Wu W, Liu Y, Huang W (2018). Verteporfin inhibits YAP-induced bladder cancer cell growth and invasion via Hippo signalingpathway. Int J Med Sci..

[150] Wu L, Yang X (2018). Targeting the Hippo pathway for breast cancer therapy. Cancers (Basel)..

[151] Jia J, Qiao Y, Pilo MG, Cigliano A, Liu X, Shao Z (2017). Tankyrase inhibitors suppress hepatocellular carcinoma cell growth via modulating the Hippocascade. PLoS ONE.

